# Boron-Doped Mesoporous Bioactive Glass Nanoparticles (B-MBGNs) in Poly(ε-caprolactone)/Poly(propylene succinate-*co*-glycerol succinate) Nanofiber Mats for Tissue Engineering

**DOI:** 10.1021/acsabm.4c01871

**Published:** 2025-06-13

**Authors:** Clara Dourado Fernandes, Sena Harmanci, Alina Grünewald, Zoya Hadzhieva, Bruno F. Oechsler, Claudia Sayer, Pedro H. Hermes de Araújo, Aldo R. Boccaccini

**Affiliations:** † Department of Chemical Engineering and Food Engineering, 28117 Federal University of Santa Catarina, Florianópolis, Santa Catarina 88040-900, Brazil; ‡ Institute of Biomaterials, Department of Materials Science and Engineering, 9171 University of Erlangen-Nuremberg, Cauerstr. 6, Erlangen 91058, Germany

**Keywords:** electrospinning, benign solvents, mesoporous bioactive glass nanoparticles, scaffolds, biodegradable polymers

## Abstract

Increased demand for advanced biomaterials in tissue engineering has driven research to develop innovative solutions based on smart material combinations. Mesoporous bioactive glass nanoparticles (MBGNs) have emerged as attractive materials because of their angiogenic and regenerative properties. This study explores the incorporation of boron-doped mesoporous bioactive glass nanoparticles (B-MBGNs) into poly­(ε-caprolactone) (PCL) and poly­(propylene succinate-*co*-glycerol succinate) (PPSG) fibers to enhance their biodegradation and bioactivity. B-MBGNs were synthesized via a microemulsion-assisted sol–gel method and characterized through morphology, pore size distribution, composition, and surface area. PCL/PPSG nanofibers were fabricated using an alternative combination of solvents, formic acid, and acetic acid. B-MBGNs were incorporated into PCL/PPSG solutions at concentrations of 5, 10, and 15 wt % and electrospun into nanofiber mats under a flow rate of 0.2 mL/h at 22 °C and 40% relative humidity, while the voltage applied at the needle tip was 18 kV and −2 kV at the rotating drum. The addition of 10 wt % of B-MBGNs resulted in nanofibers that exhibited a high degradation rate in PBS with a weight loss of 44% in 30 days, significant hydrophilicity with a contact angle of 33°, and improvements in cell viability tested with normal human dermal fibroblasts (NHDF). In addition, the study highlights the effect of the concentration of B-MBGNs on the morphology of the fibers, which can agglomerate and form undesired beads. Although the particles improved cellular activity, the changes in morphology caused tension points that reduced the elasticity of the fibers. Overall, this work contributes to the innovative use of green polyesters combined with boron ions in electrospun fibrous scaffolds, expanding the opportunities for applications in tissue regeneration, for example, to treat chronic wounds in diabetic patients.

## Graphical abstract

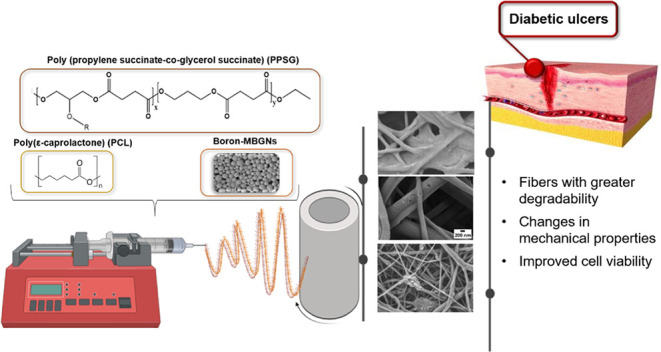

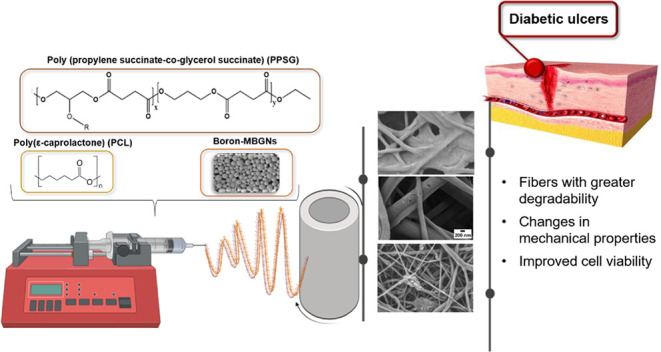

## 1 Introduction

The growing demand for advanced biomaterials capable of replacing damaged or diseased tissues has driven research in the development of novel combinations of bioactive materials. Among them, mesoporous bioactive glass nanoparticles (MBGNs) stand out for their ability to stimulate osteogenesis, induce angiogenesis, and form strong bonds with bone and soft tissues.^1,2^ Their high specific surface area and porosity enhance apatite mineralization and interactions with the biological microenvironment.^3–5^ The doping of MBGNs with therapeutic metallic ions, within the scope of ionic medicine, has emerged as a promising strategy to enhance their biological functionality.^6^ Among the dopants studied, boron has gained attention due to its positive effects on cell proliferation and the healing of chronic wounds.^4,7^ At appropriate concentrations, boron demonstrates good cytocompatibility with fibroblasts, endothelial, and neuronal cells, and promotes neovascularization *in vivo*.^4,8–12^ As a result, boron-doped MBGNs (B-MBGNs) have been developed to impart angiogenic properties and are currently explored for wound healing applications, particularly for chronic ulcers, with FDA approval for medical device use.^1,13,14^

Despite these advances in bioceramics, critical gaps remain in understanding the application of B-MBGNs in soft tissue engineering. While their therapeutic potential is well established, few studies have investigated scaffolds that serve as targeted delivery systems for these nanoparticles. For effective tissue integration, such scaffolds and nanofibers must be biocompatible, biodegradable, and exhibit mechanical properties similar to those of native tissues.^15–17^ However, the interaction between B-MBGNs and natural or hybrid polymeric matrices has not been fully elucidated, particularly with respect to cytotoxicity, degradation behavior, biocompatibility, and structural integrity.

In this context, biodegradable polymers are increasingly explored as matrices for bioactive scaffolds. The copolymer poly­(propylene succinate-*co*-glycerol succinate) (PPSG), synthesized via an enzymatic route, has emerged as an innovative alternative due to its high hydrophilicity, biodegradability, and tunable mechanical properties.^18,19^ These features make PPSG particularly promising for soft tissue applications, surpassing the limitations of widely used polymers such as poly­(glycerol sebacate) (PGS) and poly­(1,8-octanediol-*co*-citrate) (POC), which require high temperatures and toxic catalysts.^20,21^ Nevertheless, the low molar mass of PPSG limits its electrospinnability when used alone, necessitating its blending with more processable polymers, such as poly­(ε-caprolactone) (PCL).

PCL is widely used in nanofiber production due to its biocompatibility, ease of processing, and established biomedical application record.^22–24^ Previous studies have shown that its combination with glycerol-based polyesters, such as PGS and POC, improves the mechanical and degradation properties of the resulting scaffolds.^17,20,25–27^ However, most of these systems rely on toxic solvents such as chloroform and DMF, which are unsuitable for clinical applications.^28^ To overcome this limitation, alternative solvents like formic and acetic acids have been successfully employed to reduce processing toxicity.^29,30^

This study investigates, for the first time, the incorporation of B-MBGNs into PCL/PPSG nanofibers to enhance their physical, chemical, and biological properties, focusing on applications in chronic wound healing. The combination of PPSG with PCL offers a balance of processability, biocompatibility, and tunable degradation, while boron doping is explored for its angiogenic potential. Additionally, acetic and formic acids are used as alternative solvents to minimize the toxicity of the electrospinning process. Thus, we evaluated how the addition of B-MBGNs influences the structure and bioactivity of nanofibers developed in a novel PCL/PPSG polymer blend, aiming to develop bioactive scaffolds for soft tissue regeneration.

## 2 Materials and Methods

### 2.1 Synthesis of Boron-Doped Bioactive Glass Nanoparticles

Boron-doped mesoporous bioactive glass nanoparticles (B-MBGNs) with nominal composition 50SiO_2_-40CaO-10B_2_O_3_ were obtained through the microemulsion-assisted sol–gel technique described previously.^7,31,32^ Initially, 0.79 g of cetyltrimethylammonium bromide (CTAB; ≥98%, Merck, Germany) was dissolved in 36 mL Milli-Q water at 400 rpm for 30 min at 37 °C until a clear solution was obtained. Then, 11 mL of ethyl acetate (≥99.8%, Merck, Germany) was slowly added to the system at room temperature, and the mixture was stirred for an additional 30 min to ensure proper emulsification. Subsequently, an ammonia solution (28%, VWR Chemicals, France) was prepared by mixing 0.85 mL of aqueous ammonia with 19 mL of Milli-Q water, adjusting the pH to reach pH ∼ 9. This solution was then added dropwise to the reaction mixture, followed by the addition of 4.4 mL of tetraethyl orthosilicate (TEOS, 98%, Sigma-Aldrich, Germany). The reaction was maintained under stirring for 30 min before introducing the calcium and boron precursors.

For calcium incorporation, 3.67 g of calcium nitrate tetrahydrate (99.5%, VWR Chemicals, Belgium) was gradually added to the system, followed by the addition of 0.48 g of boric acid (H_3_BO_3_, ≥99.5%, Sigma-Aldrich, Germany) for boron doping. The resulting solution was continuously stirred at 400 rpm for 4 h to ensure homogeneous nanoparticle formation. The synthesized nanoparticles were collected through centrifugation at 7000 rpm for 5 min, washed twice with Milli-Q water and once with ethanol to remove residual reagents, and then dried overnight at 60 °C. Finally, the dried material was calcined at 600 °C for 6 h with a controlled heating rate of 2 °C/min to remove organic templates and stabilize the glass network. The final product was referred to as B-MBGNs.

The characterization of the resulting particles was carried out by nitrogen physisorption using the BET (Brunauer–Emmett–Teller) and BJH (Barrett–Joyner–Halenda) models to obtain the specific surface area (SSA) and the pore size distribution, being conducted using nitrogen sorption analyzer (ASAP2460, Micromeritics Instrument). In addition, with the Zetasizer Nano ZS (Malvern Instruments, United Kingdom), DLS’s zeta potential and the average size of the nanoparticles (dynamic light scattering) were obtained by suspending the samples in deionized water.

### 2.2 Enzymatic Polyesterification of PPSG

Enzymatic polyesterification was used to obtain PPSG according to the methodology established in a previous study.^19^ Briefly, the monomers succinic acid, glycerol, and 1,3-propanediol (all from Sigma) were heated to 90 °C and homogenized under constant stirring (700 rpm) for 30 min. Subsequently, 10 wt % of immobilized *Candida antarctica* enzyme was added to the system at a pressure of 0.1 atm for 24 h.

The resulting polymer exhibited a weight-average molecular weight (*M*_w_) of 11 ± 0.4 kDa, a number-average molecular weight (*M*_n_) of 3 ± 0.6 kDa, and a molar mass dispersity of 2.3, indicating a moderate molecular weight distribution. Additionally, the polymer presented a degree of polymerization of 15, a gel content of 13%, and an apparent viscosity of 384,3 cP. These characteristics contribute to its processability, degradability, and suitability for biomedical applications. As PPSG has been previously investigated, further details on its synthesis, characterization, and physicochemical properties can be found in Fernandes et al.^19^

### 2.3 Electrospinning Process

The electrospinning solutions were prepared by dissolving PCL (Sigma-Aldrich, 80 kDa) and PPSG (10 wt %) in 20% of the solvent mixture of formic acid and acetic acid in the ratio of 2:1 (v/v). The polymers were soluble at room temperature under constant stirring at 300 rpm for 12 h. After this, B-MBGNs were incorporated at 0, 5, 10, and 15 wt % (wt % by mass relative to the polymer mass). The solution was sonicated for 15 min and then electrospun using a commercial device (EC-CLI, IME Technologies, Netherlands).

Electrospinning was performed with a 21G needle at a flow rate of 0.2 mL/h with nitrogen gas supply at 8 mL/min, temperature of 22 °C, and 40% controlled humidity. The target was positioned 11 cm away. The voltage applied to the needle tip was 18 kV, and −2 kV was applied to the rotating collector drum, which operated at 1000 rpm. Sample names refer to the weight percentage of B-MBGNs used for electrospinning (e.g., PCL/PPSG refers to the blank sample without B-MBGNs, PCL/PPSG/5B-MBGNs, PCL/PPSG/10B-MBGNs, and PCL/PPSG/15B-MBGNs means 5, 10, and 15 wt % of B-MBGNs in relation to the polymers, respectively).

### 2.4 Characterization

Scanning electron microscopy (SEM) was used to evaluate the morphology of the B-MBGNs, the fiber mats, and the dispersion of the B-MBGNs in fibers. These analyses were performed using Auriga SEM instrument (Zeiss, Oberkochen, Germany). Before analysis, the samples were coated with a thin layer of gold using a coater (Q150T, Quorum Technologies Ltd., Darmstadt, Germany). The diameter of 100 fibers was measured for each study group using Image-J software.

The presence of B-MBGNs nanoparticles in the fiber mats was evaluated using energy dispersive X-ray spectroscopy (EDX) coupled to the SEM using gold coating to avoid sample degradation. EDX spectra were obtained at different points and regions of the samples to ensure a representative elemental composition analysis.

FTIR (Fourier-transform infrared spectroscopy) was performed over the wavenumber range 4000–400 cm^–1^ (Shimadzu IRAffinity-1S) using 30 spectra to evaluate the presence of B-MBGNs in PCL/PPSG/B-MBGNs fiber mats.

To evaluate the formation of hydroxyapatite (HAp) on samples immersed in simulated body fluid (SBF), results were obtained using an X-ray diffractometer (XRD, Miniflex, 600, Rigaku) at 2θ 10–80°. A dwell time of 1 min/degree with a step size of 0.02° was applied.

The degree of hydrophilicity was evaluated by measuring the contact angle between a water droplet and the electrospun fiber surfaces that remained for 3 s (DSA30, Kruss GmbH). Each sample was measured 5 times.

#### 2.4.1 Mechanical Characterization

Rectangular samples of length 20 mm × width 5 mm × thickness 0.4 mm were carefully cut and mounted in paper frames (20 mm × 20 mm) and fixed to the clamps of the testing machine (5960 Dual Column, Instron, Darmstadt). For each condition, ten specimens were tested with a load of 50 N and 1 mm min^–1^. Young’s Modulus (MPa), Ultimate Tensile Strength (MPa), and Elongation at Break (%) were determined for all study groups.

#### 2.4.2 Degradation Tests

The hydrolytic degradation of fiber (1 cm^2^ samples) mats was assessed by immersing them in phosphate-buffered saline (PBS) solution (Gibco Thermo Scientific, Schwerte, Germany) for 40 days at 37 °C, at 90 rpm in an incubator (KS 4000 i Control, IKA Werke GmbH & Co. KG, Staufen, Germany). At 5-day intervals, the mats were retrieved, rinsed with ultrapure water, and dried in a fume hood until a stable weight was achieved. The weight loss percentage was calculated using eq 1 by the difference in weight, where *W*_1_ is the initial weight of the mat, and *W*_2_ is the weight after drying postdegradation. Fiber morphology was evaluated using SEM, while chemical alterations were studied via FTIR analysis.

1 $$\mathrm{Weight loss}\;\left(\%\right)=\frac{W_{1}-W_{2}}{W_{1}}\times 100$$

#### 2.4.3 Hydroxyapatite (HAp) Formation *In Vitro*

To assess the ion release from B-MBGNs, fiber mats of 2 cm^2^ were immersed in triplicate in 3 mL of simulated body fluid buffer solution (SBF) prepared according to the protocol of Kokubo et al.,^33^ and incubated for 15 days at 37 °C at 90 rpm. At designated time points, the samples were retrieved, rinsed with ultrapure water, and dried in a fume hood until a stable weight was reached. Following the established protocol, XRD, FTIR, and SEM analyses were conducted to examine ion release and hydroxyapatite formation.^33^

#### 2.4.4 *In Vitro* Cell Viability Evaluation

Cell viability tests were performed to evaluate the *in vitro* cytotoxicity of the electrospun fiber mats. The assessment employed the WST-8 cell proliferation assay kit (CCK-8 kit, Sigma-Aldrich) using direct contact methodology [29]. Initially, fibers were secured in crown-shaped inserts fabricated from PCL. These samples were disinfected by UV exposure for 30 min on each side and then stored in sterile 24-well plates. The cells were maintained in DMEM (Dulbecco’s Modified Eagle Medium) from Gibco Thermo Scientific, Schwerte, Germany, supplemented with 10% fetal bovine serum (Corning GmbH, Wiesbaden, Germany) and 1% penicillin (Thermo Scientific, Schwerte, Germany). Culturing was done in 175 cm^2^ flasks until reaching 90% confluence within 48 h and incubated at 37 °C in a 5% CO_2_ atmosphere. NHDF (Normal Human Dermal Fibroblast) cells were seeded onto each sample at a concentration of 50,000 cells in 100 μL. Once the fibers were partially embedded in the cell-dense medium, 900 μL of complete growth medium was gently added to ensure full coverage of the fiber mats within the wells. The samples were incubated in a humidified CO_2_ incubator at 37 °C, with medium replacement occurring after 48 h.

After 48 h, cell viability was assessed using the WST-8 assay (1% (v/v)) from Sigma-Aldrich, with absorbance recorded at 450 nm via microplate reader. Cell viability (Cv) was determined according to eq 2), where *A_b_Sample* is the absorbance measured from the sample, *A_b_Blank* is the absorbance from wells without cells, and *A_b_Control* corresponds to the absorbance detected from wells without samples but with cells only:

2 $$Cv\left(\%\right)=\left(\frac{A_{b}\mathrm{Sample}-A_{b}\mathrm{Blank}}{A_{b}\mathrm{Control}-A_{b}\mathrm{Blank}}\right)\times 100$$

The cell morphology and attachment were examined using fluorescence microscopy. The cell nuclei were stained with DAPI (4’,6-diamidino-2-phenylindole) (Thermo Fisher Scientific, Germany), while the cytoskeleton was labeled with rhodamine-phalloidin (Thermo Fisher Scientific, Germany). Both were analyzed with a fluorescence microscope (Zeiss Observer D1, Carl Zeiss AG, Oberkochen, Germany), following the previously established protocol.^32^

### 2.5 Statistics

All data are expressed as mean ± standard deviation. Statistical tests were used in ORIGIN, applying one-way ANOVA for a *p*-value <0.05.

## 3 Results and Discussion

### 3.1 Characterization of B-MBGNs Nanoparticles

The synthesized B-MBGNs were characterized before fibers’ production. B-MBGNs were synthesized through the sol–gel process assisted by the microemulsion technique an effective technique for synthesizing mesoporous nanoparticles.^7^ Analyses of the B-MBGNs (Figure 1) revealed the formation of spherical and uniform particles, with a well-marked presence of Si and Ca. However, boron could not be detected due to its low emission energy and concentration in the sample.

**Figure 1:**
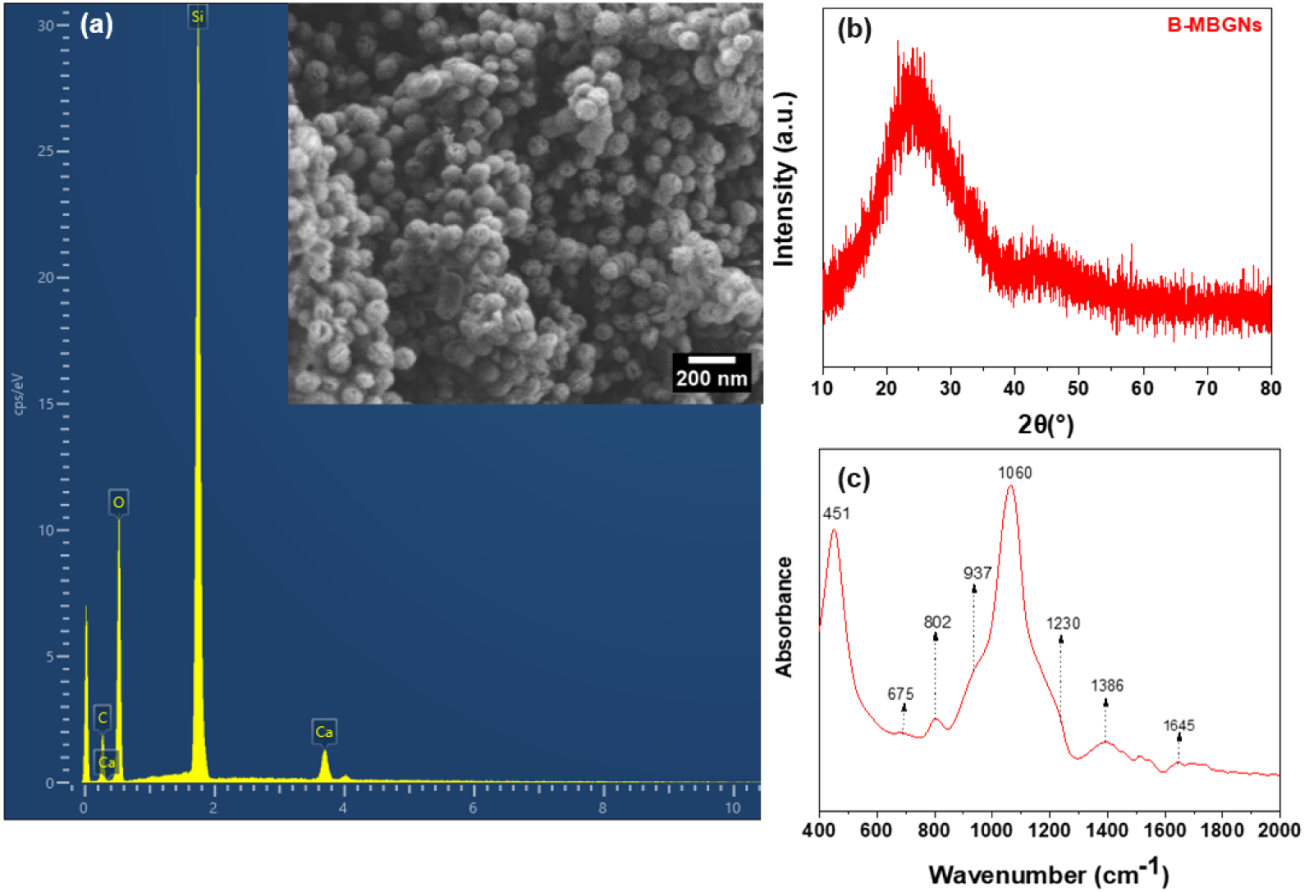
SEM image and corresponding EDX analysis of B-MBGNs (a), XRD pattern (b), and FTIR spectrum (c) of the B-MBGNs.

Table 1 presents the average particle size (nm) measured by DLS, which is 245 nm. Figure 1a shows SEM images of B-MBGNs, where the predominance of spherical particles and a relatively narrow size distribution can be observed. The average particle diameter was slightly larger than the average size of B-MBGNs produced by Zheng et al., which was 194 nm.^7^ However, B-MBGNs in the present work were significantly smaller than BG particles produced by the conventional melt-quenching method, with approximately 3.15 μm of average size.^15^ A difference was also observed between the DLS measurements and the SEM measurements. This behavior is due to the distinct principle of DLS that reflects the hydrodynamic diameter in aqueous suspension, often overestimated due to partial agglomeration. At the same time, SEM shows the dry-state morphology of better-dispersed particles.

**Table 1:** Physical Characterization of B-MBGNs

**BET Model**	**BJH Model**		**DLS**
Surface Area (m^2^ g^–1^)	Cumulative Pore Volume (cm^3^ g^–1^)	Pore Diameter (nm)	Particle Size (nm)
280	0.40	2.4	245 ± 13

Additionally, the mesoporous nature of B-MBGNs was confirmed by nitrogen physisorption analyses. The BET specific area was 280 m^2^/g, with a total pore volume of 0.40 cm^3^/g and an average pore diameter of 2.4 nm, as calculated by the BJH model. These values fall within the IUPAC-defined range for mesoporous materials (2–50 nm), confirming that the synthesized particles are indeed mesoporous. The high specific area and narrow pore diameter distribution are attributed to the role of ethyl acetate in forming stable microemulsion droplets and the cationic surfactant CTAB as a structure-directing agent, which promotes the formation of organized mesopores during the sol–gel transition.^31,34^ These results are consistent with those reported by Zheng et al.,^7^ who synthesized B-MBGNs via a similar method and obtained a specific area of ∼300 m^2^ g^–1^ and pore sizes in the range of 2–5 nm. Moreover, the study by Hosseini et al.^35^ demonstrated how specific functionalization with APTES significantly reduced the accessible specific area and pore volume of mesoporous silica nanoparticles, from 788 m^2^ g^–1^ and 0.46 cm^3^ g^–1^ to 194 m^2^ g^–1^ and 0.10 cm^3^ g^–1^, respectively, due to partial blocking of the mesoporous channels. These findings further underscore the structural sensitivity and tunability of mesoporous systems, reinforcing that our synthesized B-MBGNs share the characteristic textural properties of mesoporous materials.

The XRD results presented in Figure 1b confirm the amorphous nature of B-MBGNs, evidenced by the lack of crystalline peak characteristic peak at 23°.^36^ The absence of diffraction peaks associated with boric acid crystals indicates a uniform integration of boron into the B-MBGNs. This result confirms that B-MBGNs can be synthesized via the microemulsion method, with boric acid serving as a suitable forerunner for boron incorporation into the silicate structure.

FTIR results of nanoparticles can be seen in Figure 1c, in which the resonances around 451 and 802 cm^–1^ refer to the symmetric bending and stretching vibrations of Si–O–Si.^37^ The vibration around 1060 cm^–1^ reflects the asymmetric stretching of Si–O–Si tetrahedra, while the shoulder observed at 1230 cm^–1^ refers to the stretching of SiO_4_.^36^ The presence of molecular water induced the peak at 1645 cm^–1^,^36^ which reflects silica’s presence in the nanoparticles’ composition.^38^

B-MBGNs show a shoulder at 937 cm^–1^ attributed to the B–O stretching vibration of BO_4_ units.^37,39^ Additionally, bands at 1386 and 675 cm^–1^ represent B–O–B vibrations of [BO_3_] units in stretching and B–O–Si bond, respectively.^40,41^ Thus, the FTIR spectrum confirms the presence of boron and the formation of bonds (B–O–Si).^7^ The B_2_O_3_ present in silicate BG can cause the three-dimensional structure of Si–O–Si to break down and form tridentate chain triangles (BO_3_). However, the FTIR results indicate that boron in B-MBGNs exists mainly as B–O–B bonds. At the same time, B–O–Si bonds are not present in high concentrations, possibly due to the instability of Si–O–B bonds.^36,40^

These results confirm that the synthesized B-MBGNs possess key features of mesoporous bioactive glass nanoparticles, including nanoscale morphology, amorphous glass structure, high specific area, mesoporous pore distribution, and boron incorporation.

### 3.2 Chemical Characterization of PCL/PPSG/B-MBGNs Electrospun Mats

After preparation and characterization, the B-MBGNs were incorporated into PCL/PPSG solutions at concentrations of 5, 10, and 15 wt % of the polymer to produce electrospun fibers. After measuring the diameter of 100 fibers for each condition, the average diameter distribution is expressed in Figure 2.

**Figure 2:**
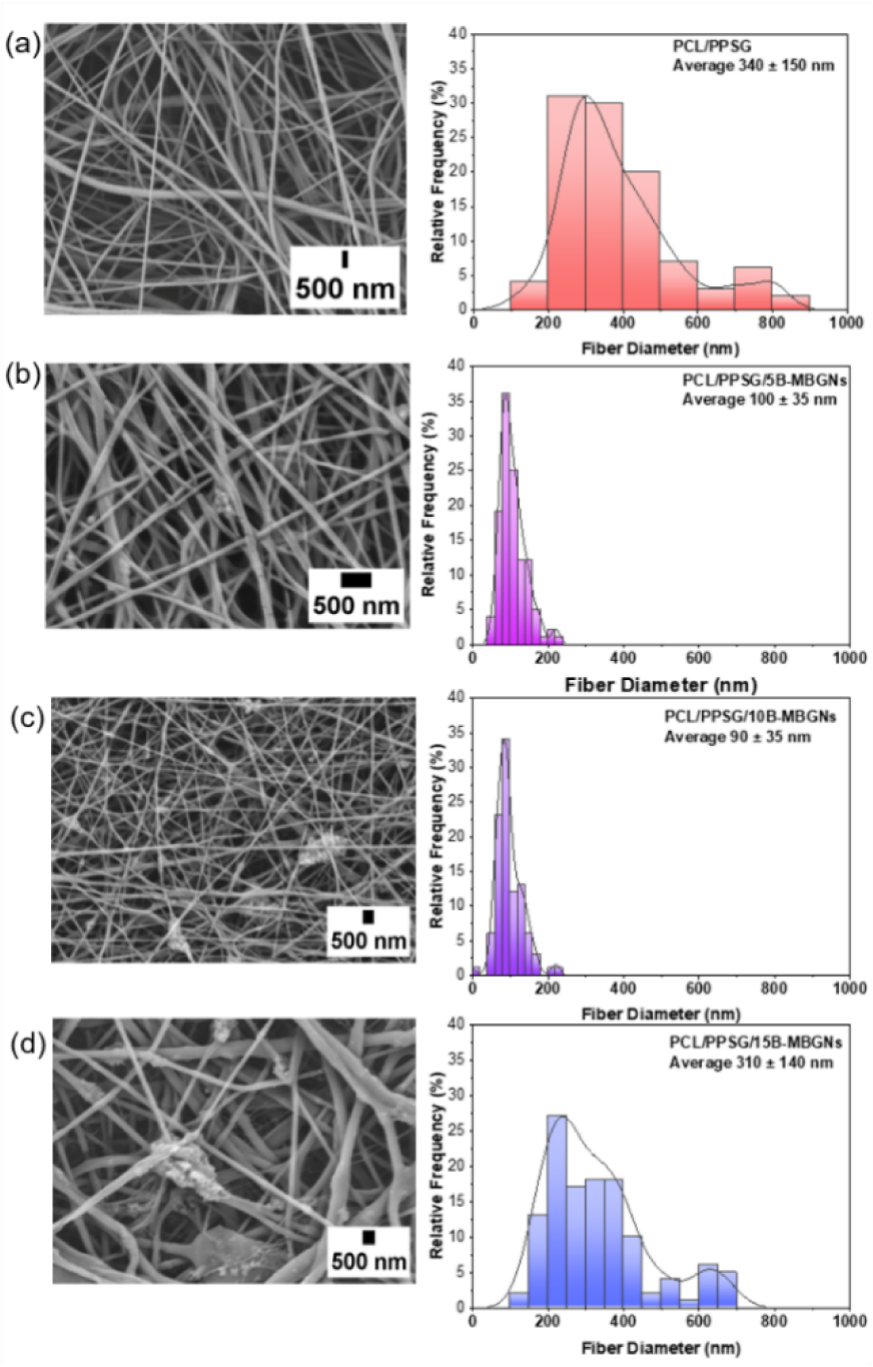
SEM micrographs of the electrospun samples and their respective size diameter distribution, without B-MBGNs (a) PCL/PPSG, and with 5 wt % (b) PCL/PPSG/5B-MBGNs; with 10 wt % (c) PCL/PPSG/10B-MBGNs; and 15 wt % (d) PCL/PPSG/15B-MBGNs.

It was demonstrated that glass particles were more evenly distributed in the less viscous solutions created by combining the benign solvents formic acid and acetic acid (2:1).^20,42^ Thus, the applied voltage of 20 kV at a distance of 11 cm proved to be suitable for the polymer solution with low viscosity. The concentration of B-MBGNs was varied to investigate its influence on the bioactivity and physical properties of the electrospun mats (Figure 2a–d).

Figure 2 also shows that nanofiber structures were formed and that the B-MBGNs influenced the fiber morphology regarding average fiber diameter. For the samples containing 5 and 10 wt % B-MBGNs, a decrease in fiber diameter was observed compared to PCL/PPSG, which is likely due to the increase in ionic conductivity caused by the addition of the nanoparticles.^43,44^ Increased conductivity enhances the electrostatic force acting on the polymer jet, leading to greater elongation and, consequently, smaller fiber diameters.

However, at a concentration of 15 wt %, a different behavior was observed. Instead of a continued decrease in fiber diameter, an increase in fiber diameter variation occurred. This effect is not primarily attributed to further increases in ionic conductivity, but rather to difficulties in nanoparticle dispersion (Figure S1). At this concentration, B-MBGNs begin to form agglomerates, as the polymer solution struggles to maintain a stable dispersion of nanoparticles.^43,45^ These agglomerates disrupt the uniform flow of the electrospinning jet, leading to jet instability and broader fiber diameter distributions. The inhomogeneous dispersion of nanoparticles at 15 wt % is supported by the presence of clusters visible in the fiber structure, confirming that excessive nanoparticle loading can negatively impact fiber morphology.

Additionally, the B-MBGNs nanoparticles should be evenly distributed throughout the electrospun mats, as this affects the scaffolds’ bioactive and mechanical properties. To verify the presence of B-MBGNs, the elemental map obtained by EDS (Figure 3) shows the homogeneous presence of Si and Ca in all the PCL/PPSG/5B-MBGNs polymer matrices.

**Figure 3:**
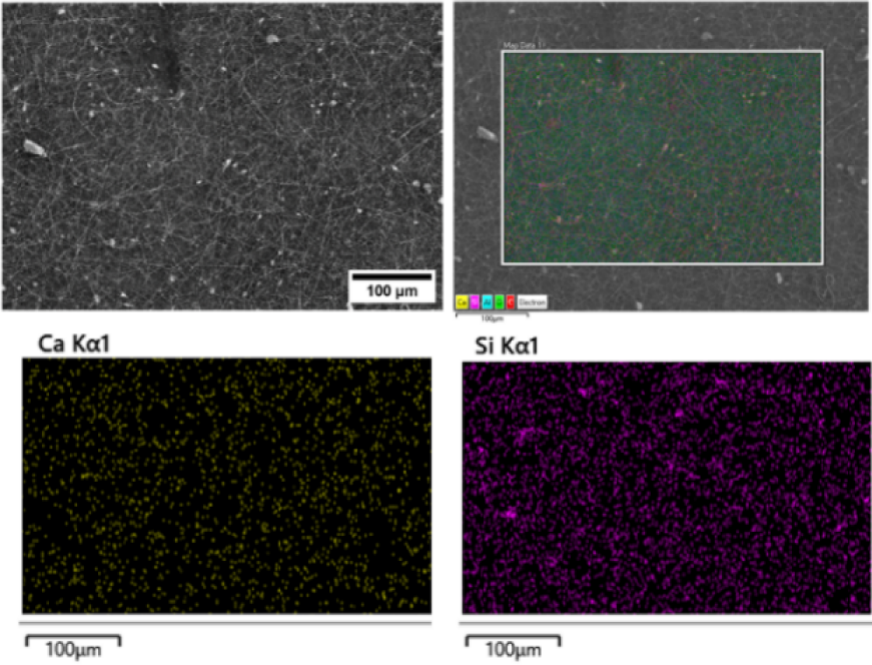
EDS mapping of electrospun nanocomposite scaffold PCL/PPSG/5B-MBGNs showing distribution of Ca and Si elements on the surface.

The FTIR spectral analysis reveals the accentuation of characteristic bands that confirm the presence of the B-MBGNs (Figure 4). The observed bands for the PCL/PPSG polymer blend are consistent with the literature on aliphatic polyesters. The prominent identified bands include the wavelengths of 2937 cm^–1^ and 2860 cm^–1^. These peaks are attributed to the asymmetric and symmetric stretching of the C–H bonds in the CH_2_.^43^ The peak at 1363 cm^–1^ corresponds to the symmetric deformation of the C–H group characteristic of aliphatic polyesters such as PCL and PPSG.^19,46,47^ The carbonyl stretching (C=O) present in PCL/PPSG indicates the presence of the ester group in the band at 1726 cm^–1^.^48^ Similarly, the symmetric and asymmetric stretching of the C–O–C bonds in both PCL and PPSG is identified at 1240 cm^–1^ and 1165 cm^–1^.^46^

**Figure 4:**
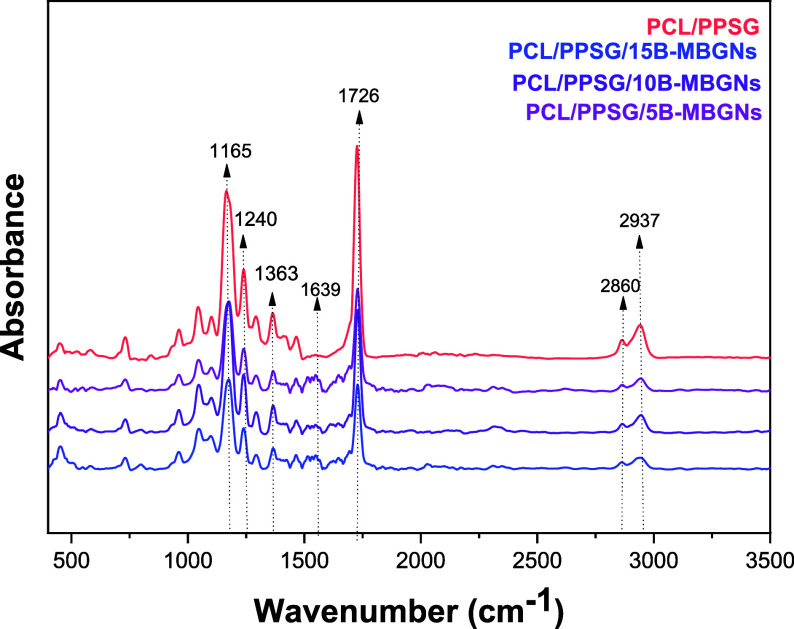
FTIR spectra of fiber mats in the 400 to 3500 cm^–1^ wavenumber range.

In samples with B-MBGNs (PCL/PPSG/B-MBGNs), in addition to the aforementioned ones, bands with deformations, indicating the presence of bioactive glass, were found. The band at 1639 cm^–1^ can be attributed to the presence of adsorbed water, indicating the hygroscopic nature of the bioactive glass nanoparticles.^49,50^ Similarly, the band’s broadening at 450 cm^–1^ can be associated with the bending vibrations of the Si–O–Si bonds in BG, indicating the amorphous silicate structure of the material.^47^ Conversely, the characteristic peak of the B–O stretching vibrations in BO_4_ units, which could confirm the incorporation of boron into the glass structure at 930 cm^–1^, is overlapped by the stretching vibrations of the C–C and C–O bonds of the PCL/PPSG polyesters.^47^

### 3.3 Mechanical Properties

Tensile tests indicate that incorporating B-MBGNs into electrospun PCL/PPSG fibers significantly impacted their mechanical properties, which can be related to the distribution and interaction of BG nanoparticles with the polymer matrix.

The Young’s modulus increased simultaneously with the concentration of B-MBGNs, indicating, as expected, that the presence of nanoparticles exerts a stiffening effect. On the other hand, although the UTS (MPa) also increased initially (0.8 ± 0.1 MPa and 1.2 ± 0.1 MPa in samples PCL/PPSG, PCL/PPSG/5B-MBGN, respectively), there was a slight decrease with higher B-MBGNs concentrations (PCL/PPSG/10B-MBGNs, PCL/PPSG/15B-MBGNs) possibly due to nanoparticle agglomeration, which can create stress concentration points. The elongation at break consistently decreased with increasing B-MBGNs concentration, reflecting the reduction in deformation capacity of the fibers before fracture (Table 2).

**Table 2:** Mechanical Properties of Composite Fibers with B-MBGNs

**Sample**	**Young’s Modulus (MPa)**	**UTS (MPa)**	**Elongation at Break (%)**
PCL/PPSG	3.8 ± 0.3	0.8 ± 0.1	55.2 ± 1.9
PCL/PPSG/5B-MBGNs	4.7 ± 0.3	1.2 ± 0.1	49.5 ± 4.3
PCL/PPSG/10B-MBGNs	8.3 ± 0.5	1.1 ± 0.1	47.3 ± 7.1
PCL/PPSG/15B-MBGNs	11 ± 1.2	1.0 ± 0.1	37.0 ± 6.3

These results are in agreement with previous studies, where adding rigid particles to a polymer matrix generally results in a more rigid and resistant composite but reduces ductility. For example, Luginina et al.^32^ reported an increase in Young’s modulus from 3.8 to 4.5 MPa when glass particles were added to the PCL/PGS polymeric blend. At the same time, elongation at break decreased (219% to 117%), in agreement with our findings. Similarly, Liverani et al.^29^ observed that BG particles also reduced the deformability of PCL fibers blended with chitosan (CS) (PCLCS/mBG) when compared to samples in the absence of particles, going from 37 to 17 MPa.

Therefore, a B-MBGNs concentration of around 10% may be ideal for applications that require a combination of stiffness and strength with some residual flexibility. Higher concentrations may be reserved for applications where stiffness is the main priority, but with the awareness that ductility will be significantly reduced.

### 3.4 Wettability and Degradation in PBS Solution

Hydrophilicity is a critical factor for materials applied in tissue regeneration since more hydrophilic scaffolds favor cell adhesion and cell morphology development. In this analysis, it was observed that PPSG improved the hydrophilicity of the fiber, which caused the reduction of the contact angle from 117° for pure PCL fibers to around 26° for PCL/PPSG fibers, as can be observed in Figure 5. Previous studies have already reported that blends made with hydrophilic polymers (gelatin, PMMA, PGS, and collagen) can cause modulation of the hydrophobicity of the predominant polymer (PCL, PLA).^52,53^ Furthermore, the presence of B-MBGNs did not induce a statistically significant difference in the hydrophilicity of the mats.

**Figure 5:**
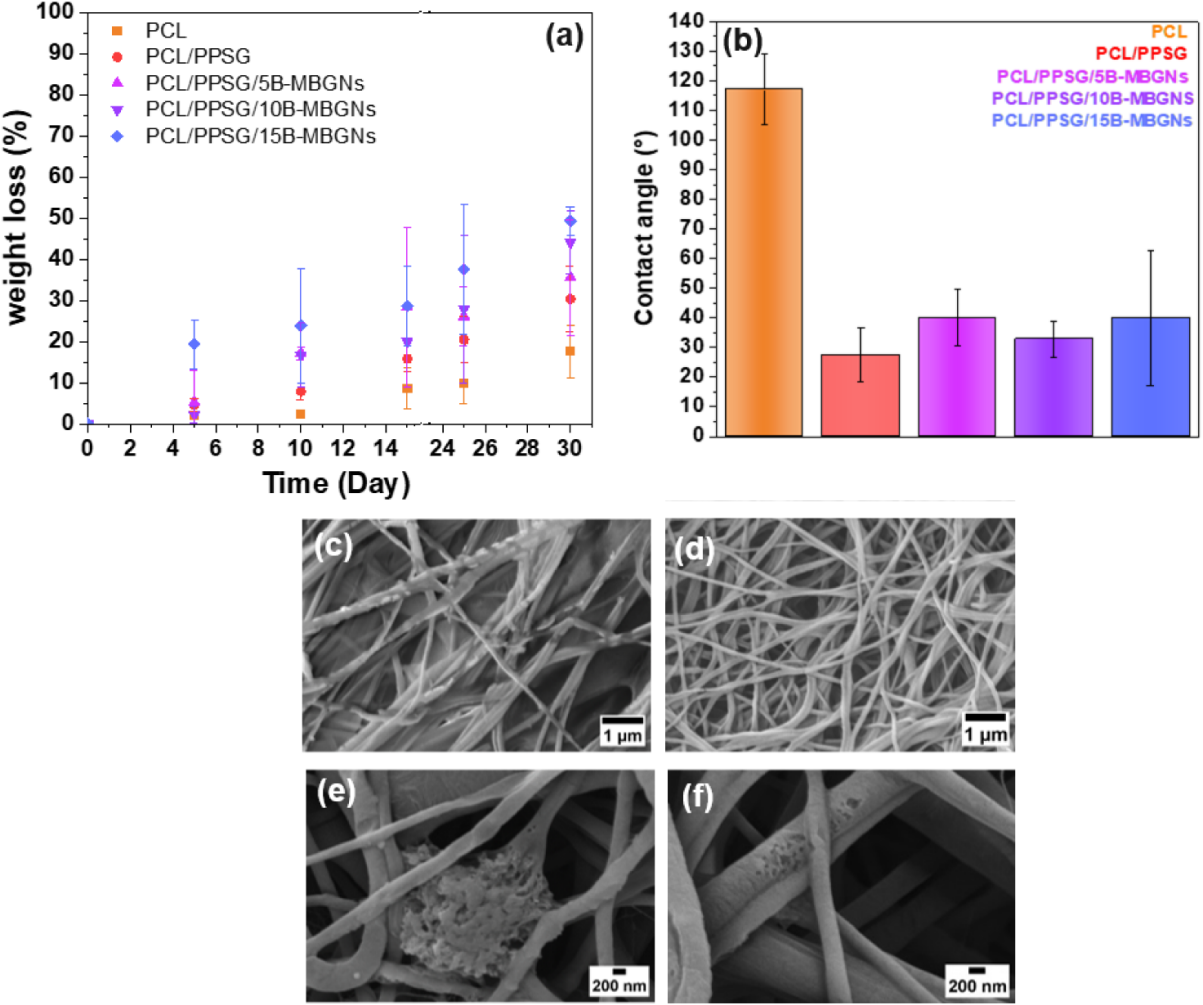
Weight loss during immersion of the fiber mats in PBS at 37 °C for 30 days (a), contact angle of electrospun mats with varying amounts of B-MBGNs (b), and SEM images of samples after 30 days of immersion in PBS: PCL/PPSG (c); PCL/PPSG/5B-MBGNs (d); PCL/PPSG/10B-MBGNs (e); PCL/PPSG/15B-MBGNs (f).

Despite the significant reduction in hydrophobicity, contact angle measurements did not show a clear trend with increasing nanoparticle concentration. This behavior is likely attributed to variations in surface roughness and the nonuniform distribution of B-MBGNs on the fiber surface, as evidenced by SEM and EDS analyses (Figures 2 and 3). Although no statistically significant trend was observed in the contact angle results, all conditions tested demonstrated a substantial increase in wettability compared to pure PCL fibers. This increase was sufficient to influence the degradation behavior of the electrospun mats in PBS, as reflected in the mass loss analysis (Figure 5a).The topographic interface generated by B-MBGNs is relevant for biodegradation.^54^ Previous studies have reported that biomaterials undergo progressive changes in their properties over time. In the case of the PCL/PPSG polymer blend, hydrolysis of the ester bonds is expected to result in mass loss.^55,56^

The mass loss of the fibers immersed in PBS (Figure 5a) points to the strong influence of the hydrophilicity of PPSG. PCL fibers presented a lower degradation rate, while the rapid degradation of PCL/PPSG/B-MBGNs fiber mats is more pronounced due to the hydrolytic breakdown of ester linkages in the material components.^57^ Microscopic analyses show increased fiber roughness, evidenced by pore and crack formation likely due to polymer and B-MBGNs leaching (Figure 5). For instance, the leaching of agglomerated inorganic particles in the mats led to the formation of tiny pores in the sample PCL/PPSG/15B-MBGNs.

Additionally, weight loss measurements confirmed the accelerated degradation of PCL/PPSG/B-MBGNs blends compared to pure PCL/PPSG. Overall, the PCL/PPSG blends exhibited a mass loss of approximately 40% after 30 days, highlighting their rapid degradation compared to other natural polymer blends.^22^ It is important to note that all samples showed high standard deviations in the results, indicating significant variability in the measurements. This variability can be attributed to intrinsic factors; thus, the results should be interpreted cautiously.

Moreover, the results indicate that PCL/PPSG/B-MBGNs fibers have potential for biomedical applications where controlled degradation and high hydrophilicity are desirable. The rapid degradation and increased hydrophilicity can favor controlled drug release and cell interaction, making these fibers promising candidates for tissue engineering and other biomedical devices. Therefore, the incorporation of B-MBGNs in PCL/PPSG fibers modulates both physical properties and degradation rates, offering a versatile platform for the development of advanced biomaterials.

### 3.5 *In Vitro* Acellular Bioactivity

PCL/PPSG fibers containing B-MBGNs were characterized after 14 days of immersion in a simulated body fluid (SBF) solution. XRD analysis identified hydroxyapatite formation in all samples regardless of the B-MBGNs concentration (Figure 6), while FTIR analysis (Figure 7) revealed the presence of peaks characteristic of interactions between the components. SEM images (Figure 7) confirmed hydroxyapatite formation, corroborating the FTIR and XRD data.

**Figure 6:**
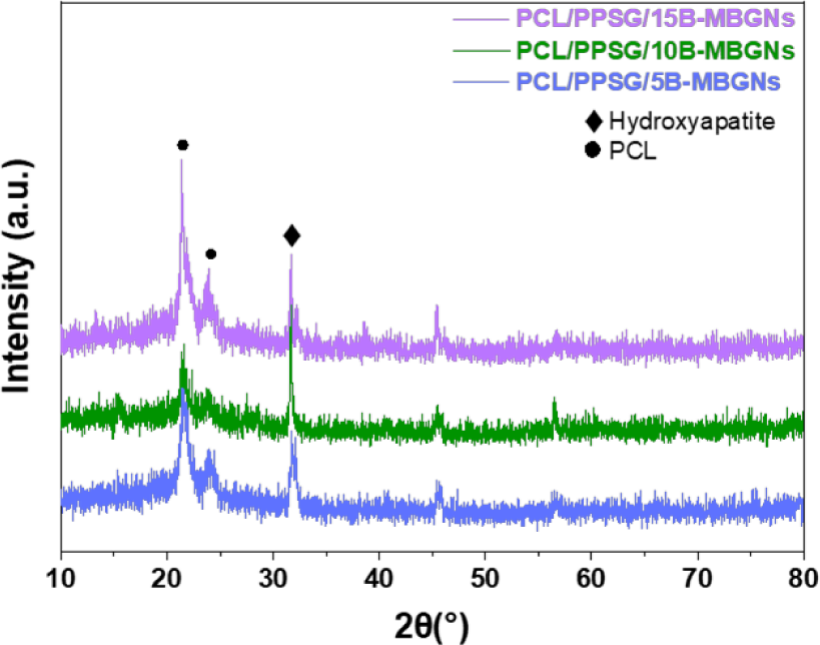
XRD patterns of B-MBGNs electrospun fibers incubated in SBF solution over 14 days. The peaks at 21.4° and 23.8° indicate the crystal structure of PCL (●), and the peak at 32.2° indicates the presence of hydroxyapatite (⧫).

**Figure 7:**
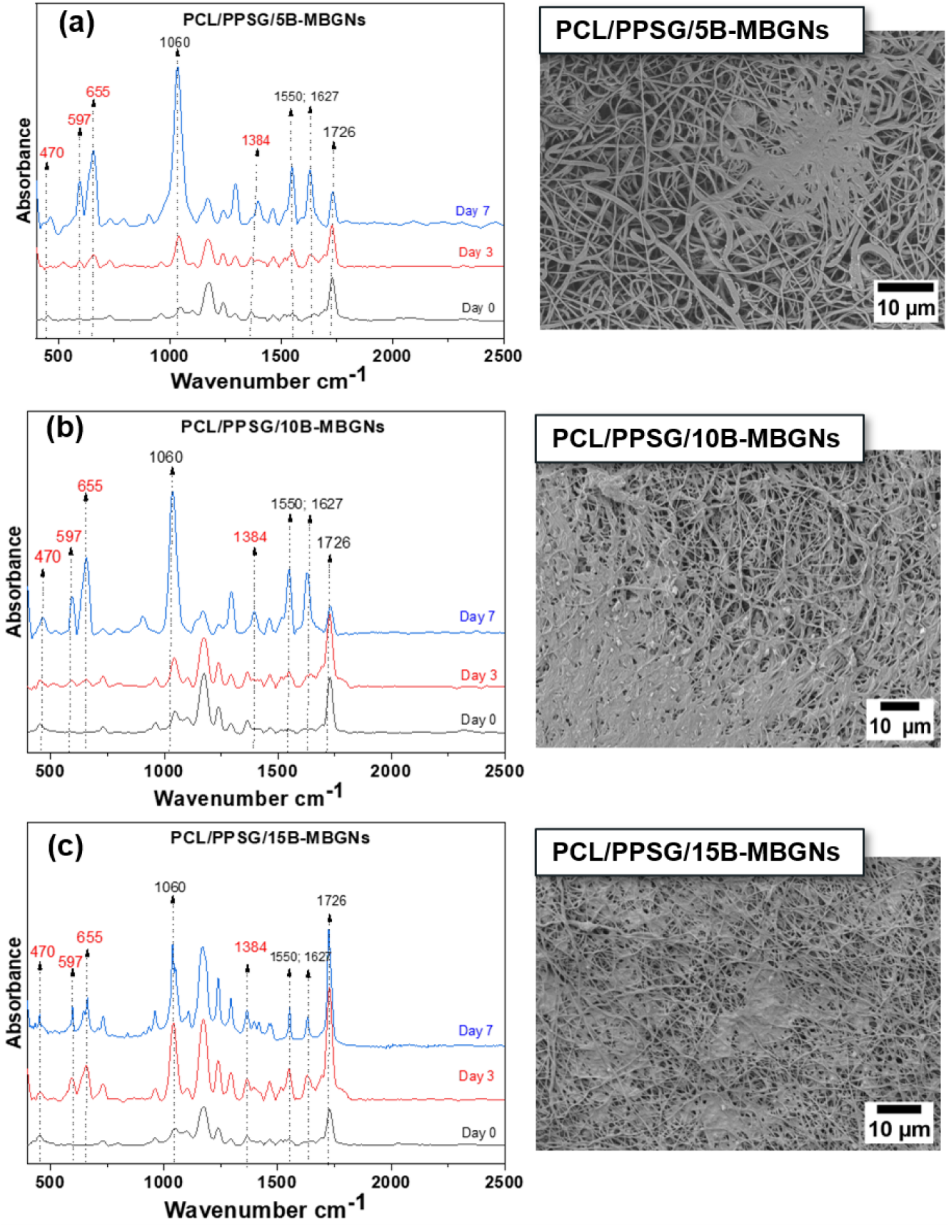
FTIR analysis of electrospun PCL/PPSG fiber mats containing 5% of B-MBGNs (a), 10% of B-MBGNs (b), and 15% of B-MBGNs (c) immersed in SBF solution over 7 days and their respective SEM images.

The XRD patterns of PCL/PPSG/B-MBGNs fibers with different concentrations of B-MBGNs (5%, 10%, 15%) showed peaks at 2θ 21.3°, 23.8°, 31.7°, and 32.2° at the end of the immersion time (Figure 6). The peaks at 31.7° and 32.2° are characteristic of hydroxyapatite. They may be associated with different phases of calcium phosphate, including β-TCP (calcium triphosphate), indicating the formation of a bioactive phase in the B-MBGNs samples.^15,58^ These results confirm that the B-MBGNs are capable of inducing HAp formation.

Concerning the FTIR results of the PCL/PPSG/B-MBGNs samples, additional peaks were observed at 1639 cm^–1^, 930 cm^–1^, and 450 cm^–1^ after immersion in SBF. The peak at 930 cm^–1^, attributed to the B–O stretching vibration, and the peak at 450 cm^–1^, associated with Si–O–Si bending vibrations, indicate the incorporation of B-MBGNs into the polymeric matrix.^7^ The decrease of the peak at 1726 cm^–1^ suggests interactions between the bioactive glass nanoparticles and the PCL/PPSG, possibly due to the formation of new chemical bonds.

SEM images confirmed the presence of HAp on the fiber surfaces after immersion in SBF (Figure 7). All PCL/PPSG/B-MBGNs samples showed crystalline structures typical of HAp, corroborating the XRD results. HAp formation was observed regardless of boron concentration in the bioactive glass, suggesting that even low B-MBGNs concentrations effectively promote bioactivity.

The results indicate that PCL/PPSG fibers doped with B-MBGNs can induce HAp formation after immersion in SBF, confirming the material’s bioactivity. FTIR analysis showed that the characteristic peaks of PCL/PPSG were maintained, while new peaks related to B-MBGNs were identified, indicating interactions between the components. The formation of HAp was confirmed by XRD and SEM, demonstrating that adding B-MBGNs does not compromise its ability to form bioactive phases.

The additional peaks at 930 cm^–1^ and 450 cm^–1^ in the samples containing B-MBGNs suggest the effective integration of the nanoparticles into the PCL/PPSG matrix and the potential creation of new active sites for HAp nucleation.^32^ The decrease of height in the peak at 1726 cm^–1^ can be attributed to the interaction between PCL/PPSG and the bioactive glass particles, indicating a modification in the polymeric matrix that favors bioactivity.^59^ The formation of HAp, confirmed by the peaks at 31.7° and 32.2°, is a clear indication of the surface bioactivity provided by B-MBGNs.^58,60,61^ Thus, PCL/PPSG/B-MBGNs fibers are promising for bone tissue engineering, providing a matrix that supports HAp formation and possibly improves tissue regeneration.

### 3.6 Cell Viability

Cell viability of PCL/PPSG/B-MBGNs electrospun scaffolds was evaluated *in vitro* for 7 days using NHDF fibroblast cells. WST-8 analysis demonstrated cell viability through metabolic activity. Figure 8 shows cell proliferation in all samples, indicating that the material is not cytotoxic. Although the samples with low concentrations of B-MBGNs (PCL/PPSG/5B-MBGNs and PCL/PPSG/10B-MBGNs) did not present a statistically significant difference between them, it was observed that all fibers composed of PCL/PPSG/B-MBGNs had greater cell proliferation than PCL fibers.

**Figure 8:**
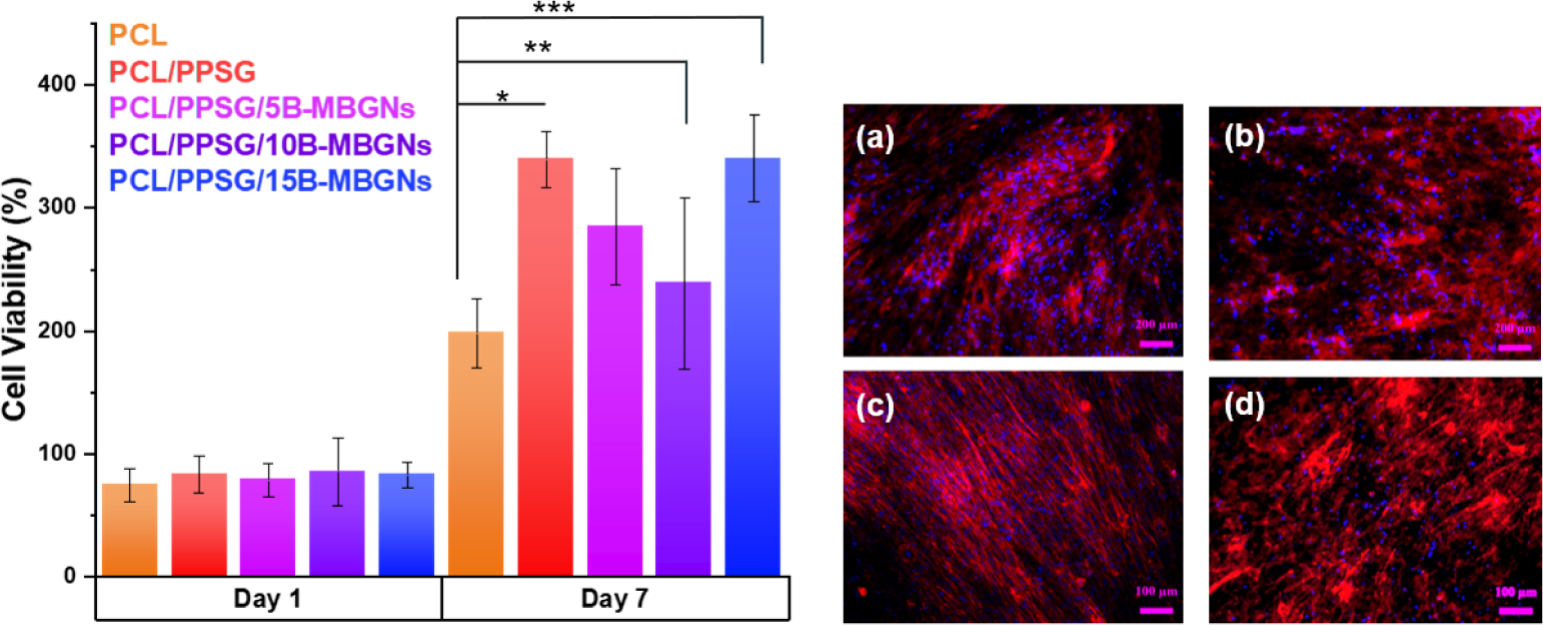
Metabolic activity measured by WST-8 analysis at 450 nm for all seedings (*p < 0.05). Fluorescence images of fibroblasts on fibers after 1 day: PSC/PPSG/5B-MBGNs (a); PSC/PPSG/10B-MBGNs (b); PSC/PPSG/15B-MBGNs (c); PSC/PPSG (d).

The results are consistent with data reported by Ege et al.^62^ who observed that high concentration of bioactive glass doped with Boron interferes with the viability of NHDF cells. Under these findings, other studies have observed a recovery in cell viability over time,^63,64^ which aligns with trends observed in our PCL/PPSG/B-MBGNs, particularly at 15% concentration. This behavior is further supported by findings from Zheng et al.,^7^ who showed no reduction in ST2 cell viability at B-MBGN concentrations up to 10 mg/mL using indirect WST assays. Likewise, Luginina et al.^32^ observed no cytotoxicity in electrospun mats with 30 wt % borosilicate bioactive glass particles using direct contact assays. Considering that the maximum concentration used in our scaffolds was 15 wt %, the data suggest that despite leaching during degradation tests, the NPs do not present any cytotoxic effect and our system operates within a biocompatibility range.

Fluorescent images support the metabolic activity results obtained by the WST-8 assay. The cell nucleus is pigmented blue (DAPI), and the actin filaments are stained red (phalloidin) (Figure 8). All samples presented cells with well-spread morphology and a robust cytoskeletal organization, indicating an environment conducive to long-term cell proliferation. Release of ions should be investigated in future work by ICP (Inductively Coupled Plasma), in order to establish a correlation between B-MBGNs concentration and fiber surface area with cell viability. The preliminary results presented in this work confirm that B-MBGNs are suitable for obtaining bioactive PCL/PPSG fibers.

## 4 Conclusions

The study showed the development of boron-doped mesoporous bioactive glass nanoparticles (B-MBGNs) incorporated into PCL/PPSG nanofiber mats. The PCL/PPSG fibers exhibited a high rate of degradability, hydrophilicity, and biocompatibility, making them suitable candidates for regenerative applications. With the incorporation of 5 and 10 wt % B-MBGNs in the PCL/PPSG solution, the average diameter of the fibers was significantly reduced, probably due to the effect of glass nanoparticles on the conductivity of the polymeric solution during electrospinning. Importantly, low concentrations of B-MBGNs preserved the mechanical integrity of the fibers, and no cytotoxic effects were observed in fibroblast cultures, confirming the biocompatibility of the nanocomposite system. The combination of PCL/PPSG with B-MBGNs yielded a multifunctional scaffold with favorable physicochemical and biological properties, supporting its potential for applications in soft tissue engineering. These findings highlight the synergistic effect of boron-doped mesoporous glass and polymeric nanofibers using a copolymer synthesized by enzymatic ring opening polymerization, which can improve the structural performance, cellular compatibility, and biosorption of the scaffolds in soft tissues.

Future work will explore fiber alignment to improve mechanical anisotropy and cellular guidance. In addition, quantitative studies of boron ion release using ICP-OES and direct measurements of solution conductivity are suggested to elucidate further the role of B-MBGNs in modulating fiber morphology and biological activity.

## Supplementary Material



## Notes

SEM images of PCL/PPSG/B-MBGNs polymer solutions containing 5, 10, and 15 wt % B-MBGNs (Figure S1), illustrating increasing nanoparticle agglomeration with higher B-MBGN content; SEM image of isolated B-MBGNs nanoparticles before incorporation into the polymer matrix (Figure S2) (PDF)
