# Supplementary material for: Boron-Doped Mesoporous Bioactive Glass Nanoparticles (B-MBGNs) in Poly(ε-caprolactone)/Poly(propylene succinate-co-glycerol succinate) Nanofiber Mats for Tissue Engineering
Source: ACS Appl Bio Mater. 2025 Jun 13;8(7):5557–67. doi: 10.1021/acsabm.4c01871 (PMC12284861; doi:10.1021/acsabm.4c01871)
Supplement: Supplementary file 1 [file mt4c01871_si_001.pdf]

Supporting Information.

Boron-doped Mesoporous Bioactive Glass Nanoparticles (B-MBGNs) in (Poly(ε-caprolactone)/Poly (propylene succinate-co-glycerol succinate) Nanofiber Mats for Tissue Engineering

Clara Dourado Fernandes[a,b] *, Sena Harmanci [b], Alina Grünewald [b]; Zoya Hadzhieva[b], Bruno Francisco Oechsler [a], Claudia Sayer [a], Pedro H. Hermes de Araújo [a] *, Aldo R. Boccaccini[b]*

[a] Department of Chemical Engineering and Food Engineering, Federal University of Santa Catarina, 88040-900, Florianópolis, SC, Brazil.

[b] Institute of Biomaterials, Department of Materials Science and Engineering, University of Erlangen-Nuremberg, Cauerstr. 6, 91058 Erlangen, Germany

*Corresponding authors

clara.dourado.fernandes@fau.de
pedro.h.araujo@ufsc.br
aldo.boccaccini@fau.de

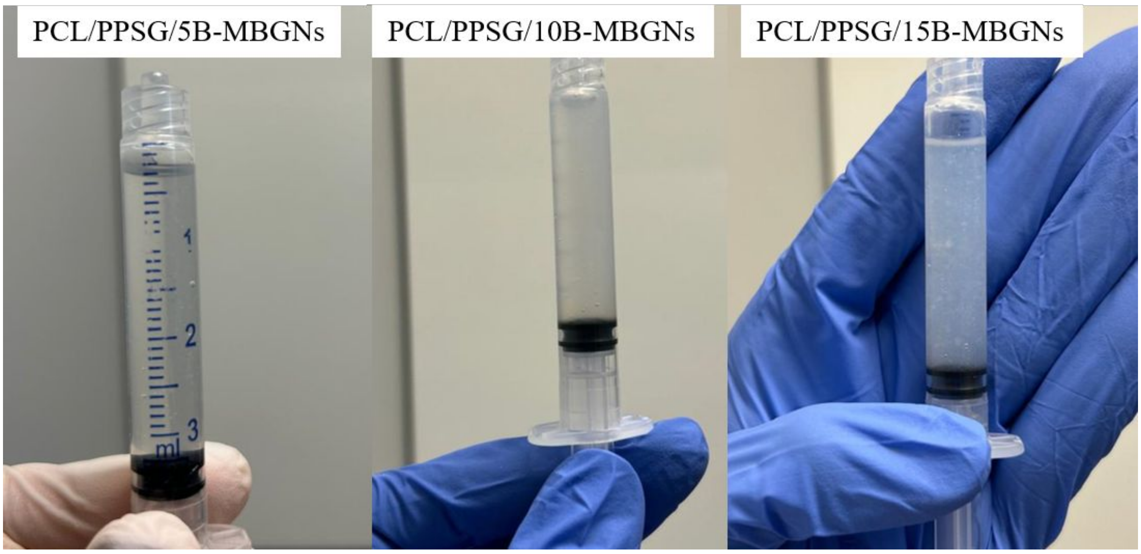

Figure S1. Polymer solutions of PCL/PPSG/B-MBGNs with 5, 10, and 15 wt% B-MBGNs, showing increased nanoparticle agglomeration at 15 wt%.

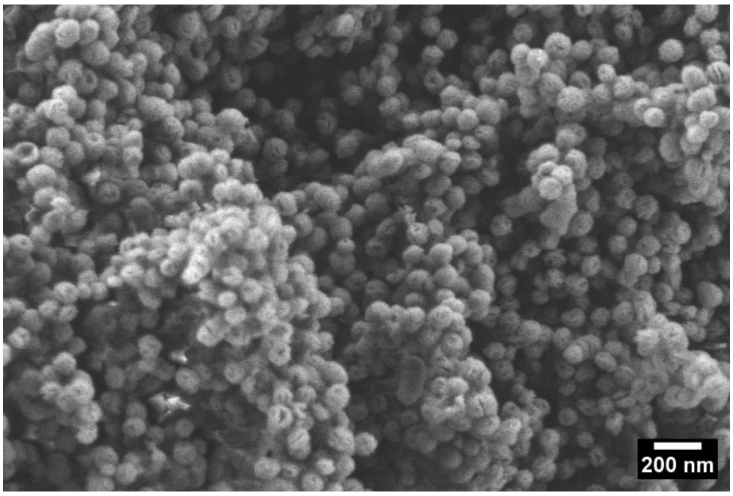

Figure S2. SEM image of the dispersed B-MBGNs nanoparticles.
